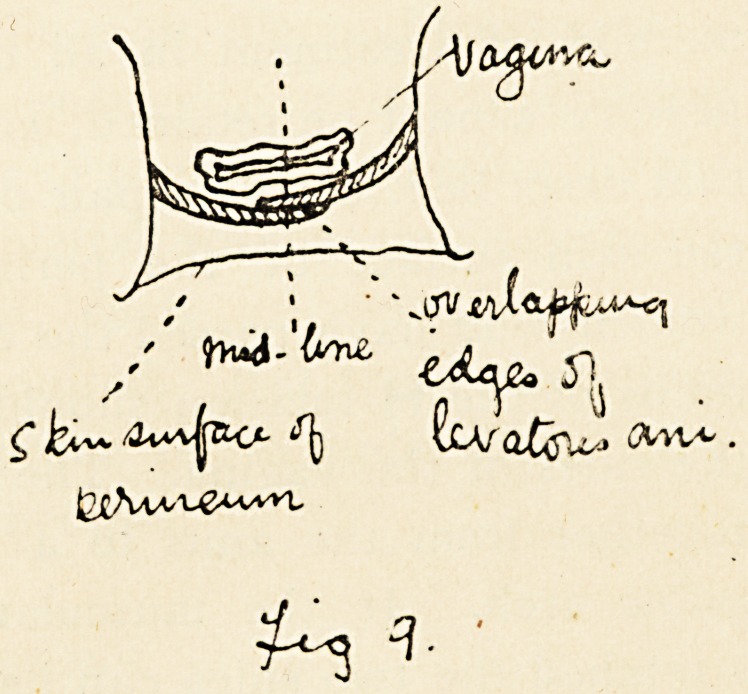# The Relation of the Pelvic Floor to Pelvic Displacements and Pain in the Female

**Published:** 1906-06

**Authors:** Ernest W. Hey Groves

**Affiliations:** Assistant-Surgeon to the Bristol General Hospital; Demonstrator of Anatomy at University College, Bristol


The Bristol

Medico-Chirurgical Journal.

"*Scire est nescire, nisi id me*
*Scire alius sciret.*"

JUNE, 1906.

### THE RELATION OF THE PELVIC FLOOR TO PELVIC DISPLACEMENTS AND PAIN IN THE FEMALE.

BY

Ernest W. Hey Groves, M.D., M.S., F.R.C.S.,

*Assistant-Surgeon to the Bristol General Hospital;*
*Demonstrator of Anatomy at University College, Bristol.*

A vast amount of literature has been written about pelvic pain and uterine displacements, and an endless number of instruments and operations have been devised for the treatment of these conditions; and the more is written the more complicated and obscure does the subject become, because so many of the theories and methods of treatment are opposed to one another. It might indeed be said that as regards the symptoms caused by minor displacements of the uterus and the best methods of treatment nothing is definitely established. Some authorities say that flexions of the uterus matter nothing, but that versions require treatment. Others consider the flexions are the conditions to be attacked, and have proposed that the crooked uterus should be kept straight by an intra-uterine stem. Others, again, find in the operation of sewing the uterus to the abdominal wall the cure for most displacements. And lastly there are those who, in the absence of active disease, never operate for the relief of mere pain, and find in the pessary the remedy for all these ills: and in this class must be reckoned the vast majority of practitioners who fit a pessary for every woman who has a displacement, and if it suits her there it stays for months or years; and if it does not she is fitted with a larger and yet larger size, until both doctor and patient give up in despair. In the present paper an attempt will be made to establish the principles underlying the cause of uterine and vesical displacements on the basis of simple anatomical facts, and to deduce therefrom the general rules which should determine treatment. It is obvious that a correct estimate of the factors concerned in maintaining the uterus and bladder in their normal position must be the guide to understanding their displacements.

The uterus and bladder are connected to the pelvic walls by folds of peritoneum, between which folds are contained strands of vascular connective tissue and some unstriped muscle. But they also rest securely on the muscular and fascial plane known as the pelvic floor. Further, they are intimately connected to one another and to the vaginal vault, so that each viscus derives additional attachment to the pelvis by the structures which support the other two.

**Peritoneal and "Ligamentous" Attachments.**—Behind, the peritoneum is intimately attached to the uterus, and is reflected from the cervix on to the posterior wall of the vaginal vault and thence on to the rectum, forming the recess known as Douglas's pouch. In front, where the peritoneum invests the uterus much more loosely, it leaves the body of this viscus and invests the bladder. At the sides the double layer of peritoneum forming the broad ligament stretches outwards and backwards to become continuous with the parietal peritoneum lining the pelvic walls. And besides the important bloodvessels and nerves and lymphatics which it contains, the broad ligament covers three fibro-muscular bands. The utero-sacral ligaments stretch from the cervix close to its junction with the body of the uterus, along the base of the broad ligament, round Douglas's pouch, to be attached to the third piece of the sacrum on each side of the rectum. The ovarian ligament runs from the posterior part of the cornu of the uterus to the ovary. The round ligament runs from the anterior part of the uterine cornu to the brim of the pelvis, where it emerges through the internal abdominal ring and inguinal canal to be attached to the pubes.

The pelvic floor is a muscular and fascial diaphragm which stretches across the pelvic cavity, from the pubes to the coccyx and from one ischial spine to the other. It consists of two pairs of muscles and the fascia covering them, viz. the coccygei and levatores ani. The former may be ignored, as they do not concern the present subject. The floor on which the uterus, vagina and bladder rest, is formed then, by the two levatores ani, with their covering fascia arising from the greater part of the walls of the pelvic basin and joining one another in the mid line. It is perhaps unfortunate that these muscles are so named and described as though they only lifted the anus and were therefore vertically disposed. As a matter of fact they are the representative of the flexor caudæ muscles of tailed

Fig. 1.

vertebrates, and are mainly composed of horizontal fibres which pass from the pubes to the coccyx. A continuous muscular sheet of fibres also arises from the "white line" of pelvic fascia and ischial spine, and the two muscles interlace in the perineum round the rectum and between the anus and coccyx. The pelvic diaphragm is perforated by the rectum behind and by the vagina with the neck of the bladder and urethra in front. Of the two gaps made by these viscera in the pelvic floor, that of the vagina and bladder is most important, not only because it is the larger, but because here the muscles of the two sides are completely separated in front. Fig. 1 is a diagram of a sagittal section of the pelvis, the levator ani forming a muscular sling passing from the pubes to the coccyx

Fig 2

being shaded in. Fig. 2 is a diagram of the pelvic floor showing its bony and fascial attachments and its two openings.

Now it is evident that the junction of the muscles in the perineum not only forms the centre of the pelvic floor on which rest the posterior vaginal wall and uterus, and on these the anterior vaginal wall and bladder, but it is also the chief means by which the two halves of the floor are held together. So that any laceration or stretching of the perineum will greatly weaken its support of the pelvic viscera by allowing the two sides of the pelvic floor to retract from one another. But there still remains for consideration a most important element in the construction of the pelvic floor, viz. the pelvic fascia. This forms a sheet of strong fascia which lines the inner surface of the pelvis covering the obturator internus muscle and the levator ani. It is attached above to the brim of the bony pelvis along the ilio-pectineal line. It is a single layer as far down as the white line, where it splits into two; the outer layer continues to cover the obturator internus and is attached below to the edge of the pelvic outlet, whilst the inner layer, known as the recto-vesical or visceral layer, covers the levator ani. When it reaches the point where the rectum, vagina and bladder lie on the pelvic floor it is reflected on to the walls of each.

In a transverse section of the pelvis taken parallel to the line of the axis of the pelvic inlet (Fig. 3) the bladder,

Fig 3.

vagina and rectum would appear to be lying one upon the other supported by the pelvic floor, and here the visceral layer of pelvic fascia is seen to split into three layers — an anterior or vesical which surrounds the bladder, a middle or genital which surrounds the vagina, and a posterior or rectal which surrounds the rectum. So that this fascia really slings the pelvic viscera from the pelvic brim. But of course it is easily stretched when deprived of the support of the levator muscle, if the latter is torn or separated from its fellow by the stretching of the perineum. The floor of the pelvis is efficient, therefore, only so long as its two lateral halves are closely approximated. Directly they are separated from one another, the viscera hanging only by peritoneum and fascia, soon descend through the gap. The rectum is closely connected to the coccyx, and the anterior wall of the bladder is similarly connected with the pubic arch, and therefore these will be least likely to be displaced. But the vagina, bringing with it the uterus and the posterior wall of the bladder, being farthest from the bony points on which they are slung, are displaced first and farthest.

The factors which support the bladder and uterus may be summarised as peritoneum, fibro-muscular ligaments, and the pelvic floor. Which of these is at fault when the viscera become displaced? The peritoneum may be dismissed from consideration, because it is evidently adapted by nature to stretch (as in distension of the bladder or in pregnancy), and cannot possess any rigidity. To the fibro-muscular bands, viz. the two utero-sacral ligaments and the round ligaments, much attention has been directed. The former sling the cervix up to the sacrum, and the latter hang the fundus to the abdominal parietes. But that they are of little or no mechanical importance is shown by several facts. First, they have never been shown to be at fault in even extreme cases of uterine displacement. In retroversion and descent of the uterus both ligaments are present and well developed, and it is evident that they can and do stretch to accommodate themselves to the position of the uterus. In the second place the vast majority of cases of uterine displacement, apart from inflammation and disease of the uterus, occur only after parturition. Yet pregnancy and parturition serve rather to thicken and strengthen these ligaments, and anyone may see that soon after parturition (just the time when so many displacements occur), the round and utero-sacral ligaments are at their stoutest. And thirdly, if the uterus was hung up by these ligaments (Fig. 4), the failure of which allowed its displacement, then when it descended it would push or invaginate the vaginal walls, dragging down the roof of the vagina (Fig. 5). But this never happens. It is always the vaginal wall which descends first, and the uterus follows. (Fig. 6.)

If, then, it is not the peritoneum or the ligaments which are at fault in uterine displacements, it must be the pelvic floor. And there is abundant evidence that this is so; for the great predisposing cause of uterine displacements is pregnancy, and this damages the pelvic floor, either tearing or stretching it. And the working-classes suffer much more from damaged pelvic floor because they are often only attended by a midwife, and any perineal laceration is apt to be overlooked. Also the working woman gets up and does her housework before the stretched pelvic floor has had time to recover its tone; and it is in the working-classes that uterine displacements most commonly occur. But most convincing of all is the actual

Fig 4

Fig 5

U = uterus

V = Vagina

L = Levator ani with overlying fascia

Fig 6

Fig 7

evidence of a damaged pelvic floor in cases where simple uterine displacements are present. In some there is an actual rupture of the perineum. In a great many the perineum is quite sound in appearance, but is very much relaxed. It only consists of skin and posterior vaginal wall, and has lost the support of the levatores ani, which have either been torn from it or stretched into a thin and useless sheet. It was always a mystery to me how or why a ruptured or relaxed perineum should cause uterine retroversion and descent. The normal uterus seems to be so far from the perineum, and the perineum itself is so feeble a structure. But the facts of the anatomy of the pelvic floor explain this mystery most simply. In the perineum the two halves of the pelvic floor are tied together. In deficiency of the perineum each half of the floor may be otherwise perfect; but it has no power to act as a support, but simply falls like a curtain against the side of the pelvis. So that the pelvic floor being divided into two halves, the first thing to fall through is the structure in the middle of the gap, viz. the vagina, and the vagina drags the uterus down with it. The relation of the perineum as the key to the pelvic floor may be illustrated by the formation of a "sedan chair" by two people's hands. Two persons clasp their hands, and on the hands a third person can comfortably be carried. But directly the hand grip is loosened the burden must drop to the ground, although the hands, arms and individuals are just as strong as ever.

The position and tone of the edge of each half of the pelvic floor can easily be felt at the side of the vagina. In a normal part it feels like a firm, round edge about one inch from the orifice of the vulva. The muscles of the two sides bring the lateral vaginal walls almost into contact, because they are joined together just behind the vagina in the perineum. But where the pelvic floor has been injured, even though the skin of the perineum is intact, these edges of the levatores ani can be felt to lie close against the pelvic wall and the vaginal orifice is widely open from side to side, and the vaginal wall prolapses through the enlarged orifice in the pelvic floor. To speak of a "relaxed vaginal outlet," as is generally done in these cases, is to miss the essential element in the case. If it was the vaginal outlet which was primarily at fault, then we should expect the uterus to drop down through the vagina. But it is a "relaxed pelvic outlet" through which the vagina falls and drags the uterus with it. In prolapse of the uterus the cervical part of the body is lengthened, as can be readily shown by passing a sound. This lengthening of the body of the uterus often amounts to half an inch or even one inch. When the organ has been replaced and kept in place by posture or a pessary for a short time, this increase in length is lost. This means that in prolapse the cervix is dragged down by the vagina, and as the fundus is held up by its ligaments the tension actually stretches out the cervical portion of the body. If on the other hand it was the failure of suspension of the uterus which allowed it to fall on to the vagina and pelvic floor, descent of the uterus would tend to compress the organ and make it shorter.

So far we have only considered descent of the uterus, but a few words must be said about retroversion of the uterus and of descent of the bladder. As was mentioned before, the anterior vaginal wall, the bladder and the uterus are so closely tied together by peritoneum and connective tissue, and by the visceral layer of pelvic fascia, that they tend to move together. When the anterior vaginal wall descends and drags the uterus down the latter will either slide round the posterior surface of the bladder, or else the bladder, uterus, and vaginal wall are together forced downwards. In either case, the anterior part of the bladder being fixed underneath the pubic arch, the uterus must descend in a circular path which lies parallel to the axis of the pelvis; so that retroversion and descent are merely parts of the same movement of the uterus. Of course, conditions of pelvic inflammation and actual uterine enlargement are not considered here, but simply those cases where displacement is the primary condition. And cystocele, or descent of the bladder pushing the vaginal wall in front of it, is merely a hernia of the bladder through the gap between the levatores ani.

Before applying these principles to the question of treatment a few words must be said about the pain and discomfort which accompany these conditions. In the first place it may be noticed that the pain is generally referred to two places, the back of the sacrum and the inguinal regions. Then it is very important to insist on the fact that the uterus itself is not tender. It can be firmly grasped, and a vulsellum can be driven into it without causing pain. When the vulsellum is pulled upon or the uterus moved about, then the pain arises. This shows that it is the structures upon which the uterine attachments drag that are the source of the pain; and of these the ovaries and tubes pulled on by the broad ligaments, and the nerve plexuses over which the uterosacral ligaments are attached, are the most important. And this is one more fact to show that the uterus is intended to be supported from below, and that directly it is hung from above pain is caused. The very great practical importance of all these facts becomes evident when we consider the question of treatment. It may seem a bold thing to suggest that anything new can be said on the subject of the repair of a ruptured perineum; but in spite of much that is written in text-books, one or two important points are hardly mentioned. The primary object in repairing the perineum should be the repair of the pelvic floor, *i.e.* the line of union of the levatores ani muscles. Of course, the three surfaces of the perineum—the rectum, the vagina, and the skin—must be restored if they have been injured; but however carefully this be done, if the muscular floor of the pelvis be neglected little or nothing will be gained. Such a proceeding is comparable to an operation on an umbilical hernia which consisted in sewing up the neck of the peritoneal sac and then stitching the skin edges, whilst leaving the gap in muscles and fascia widely gaping. In immediate suture of a torn perineum the ordinary through-and-through stitches generally are sufficient, because the muscle edges have not retracted; and as a matter of fact this immediate suture gives very good results. But for the plastic operation performed after the parts have cicatrised a great many complicated methods have been proposed and most elaborately-shaped flaps devised. The very multitude of methods advocated by different authorities shows that the operation has often been found unsatisfactory. And yet the principle to be aimed at is absolutely simple. Assuming for the moment that the rectum is intact, then three layers have to be found, their edges refreshed and sewn together each to each, viz. the posterior vaginal wall, the levatores ani, and the skin. The so-called flap-splitting method of Tait may or may not accomplish this. If good healing occurs a perineum in the sense of an area of skin between vagina and anus will always result; but if the edges of the muscular pelvic floor be not caught by the buried stitches, then the new perineum is quite useless as a pelvic support. Of course, the muscles are generally caught by the buried sutures when these pass deeply into the scar tissue, and then the operation may succeed; but it will succeed much more certainly if the muscle edges be clearly defined and sewn together without the intervention of any scar tissue at all. An H-shaped incision is best. The transverse cut splits the recto-vaginal septum and the vertical cuts serve to clearly bare the edges of the pelvic floor. (Fig. 8.) The redundant layer

Fig 8.

Fig 9.

of scar tissue in the posterior vaginal wall may be removed by a V-shaped incision, and the edges united by fine catgut. But this is not an essential step. The parallel borders of the levatores ani are then united as firmly, carefully and precisely as in the similar step in the radical cure of a hernia where the muscular layer of the abdominal parietes is sutured. The skin is then brought together by fine silkworm gut stitches over the muscular perineum.

In prolapse with retroversion of the uterus two methods of treatment are available. In the first place the pessary fulfils just the same rôle in this affection as does the truss in the treatment of a hernia. It is not ideal, it is not radical, but in many cases it is satisfactory. When the pelvic floor is incompetent by reason of laceration or stretching of its muscular fibres, the vagina prolapses through the gap and drags down the uterus. But if the gap be not too wide then a pessary inserted into the vagina will make it too wide to drop through the gap. In other words, the pessary will rest on the edges of the pelvic floor. (Fig. 7). But it is obvious that if the two halves of the pelvic floor be too widely divaricated they will simply lie against the pelvic walls, and however large a pessary be used it will slip out as easily as it slips in. The advantage of the Hodges pessary and all its modifications over the ring is that it is so shaped that its lower end lies further forward against the pubic arch—a point where the pelvic floor is attached and where its two halves lie close together.

The radical cure of this condition, which is far the best method of treatment in all cases and the only one when a pessary cannot be retained, consists in a similar operation to that described for the repair of a lacerated perineum, although the skin surface of the perineum may be unimpaired. In women who have passed the child-bearing period, the anterior limbs of the H incision should be extended far forward along the sides of the vagina. The levatores ani can then be sewn together from the anus to a point about an inch behind the pubic arch. If the muscular pelvic floor is very thin and stretched the two sides can be made to overlap by sewing the edge of the right muscle to the deep surface of the left, to the left of the mid-line and the edge of the left muscle to the outer surface of the right muscle to the right of the mid-line. (Fig. 9.)

For one form of prolapse the pessary is quite useless. That is, for a cystocele where the bladder forms a hernial protrusion through the anterior part of the gap in the pelvic floor, pushing the anterior vaginal wall before it. The pessary is useless because the edges of the two halves of the pelvic floor are widely separated just at the point where the pessary depends for support.

It is quite clear also that any operations, such as colporrhaphy or elytrorrhaphy, which simply remove pieces of the anterior vaginal wall, will be useless because the new vaginal wall will soon stretch in front of the herniated bladder. It is the pelvic floor which is at fault, and it is therefore the pelvic floor which must be dealt with. A transverse incision is made across the anterior vaginal wall one inch behind the urethra. The edges of the levatores ani are found at the extremities of the incision, the vagina is separated from the urethra and base of the bladder, and the two sides of the pelvic floor sewn together between the two viscera, thus forming a second perineum between the vagina and bladder. I described this operation in detail in the *Journal of Obstetrics and Gynecology*, March, 1905. In a recent paper[1] on Ventrifixation Dr. Herman said that he considered that in all displacements of the uterus which involved its descent, and especially when that descent affected chiefly the anterior pelvic viscera, viz. the anterior vaginal wall and bladder, this operation afforded the most effectual treatment.

When the uterus is itself enlarged, or its appendages diseased, and if from these causes or from pelvic peritonitis the uterus is displaced (generally retroverted without much descent), then other means of treatment, such as ventrifixation or Alexander's operation will be required. But these conditions are only referred to here in order to exclude them from consideration in the present connection.

### CONCLUSIONS.

1. That the pelvic viscera are supported chiefly by the underlying pelvic floor. 2. That the pelvic floor consists of a muscular diaphragm composed in the main part of the levatores ani and their investing fascia which actually attaches the viscera to the floor. 3. That the pelvic floor is adequately supported by its attachments to the circumference of the pelvis, but is weak in the mid-line where it is perforated by the rectum, vagina, and urethra. 4. That the efficiency of the pelvic floor depends upon the union of its two halves in the mid-line. 5. That displacements of the uterus, bladder, and vagina, apart from diseased conditions, depend upon a rupture, stretching or thinning of this median raphé. 6. That the adequate and rational treatment of these conditions consists in a repair of this muscular raphé either in the perineum or by forming a new second line of muscular union between the vagina and bladder. 7. That the use of a pessary constitutes a reasonable second

[1] Read before the Obstetrical Society in London, December, 1905.—*Tr. Obst. Soc. Lond.*, 1905, xlvii. 429.

best treatment, but that this will only be possible when the gap between the edges of the levatores ani is narrower than the diameter of the pessary. 8. That when the pelvic viscera lose their adequate support by the pelvic floor, they hang upon their peritoneal "ligaments," and this causes pain by dragging upon the ovaries, tubes, and pelvic nerves.

## Figures and Tables

**Fig. 1. f1:**
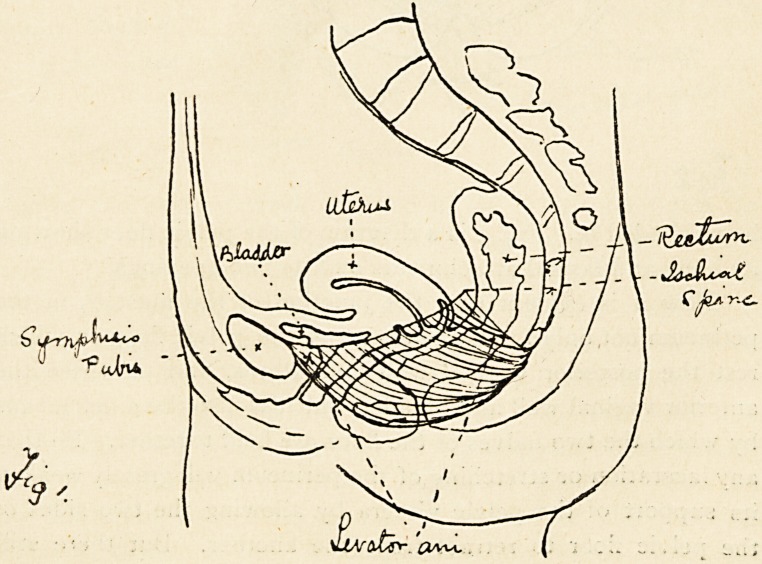


**Fig 2 f2:**
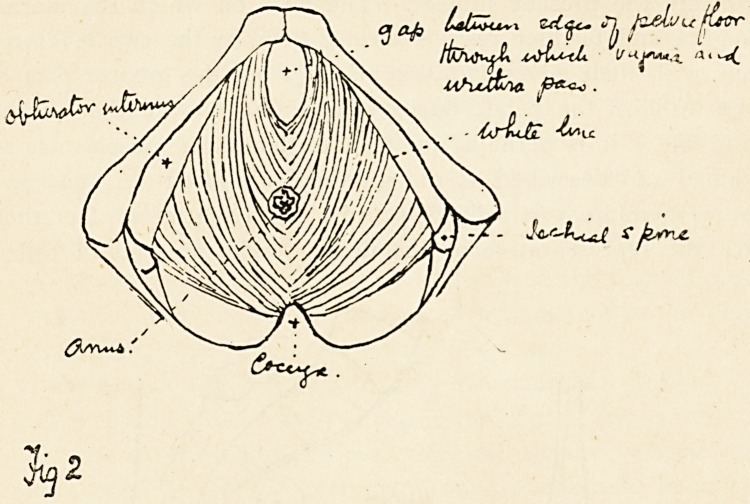


**Fig 3. f3:**
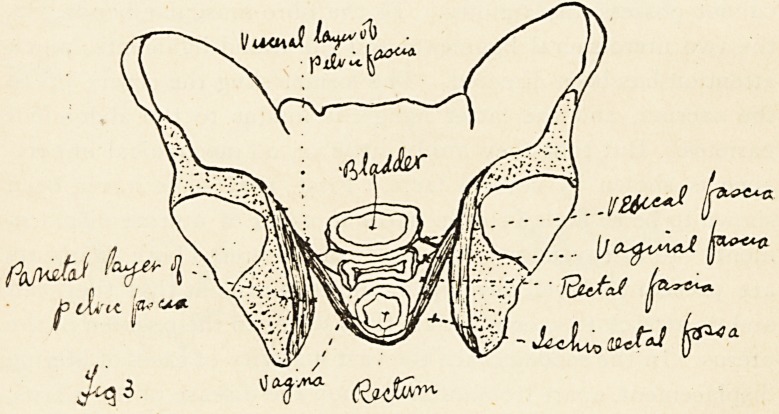


**Fig 4 Fig. 5 Fig 6. Fig 7. f4:**
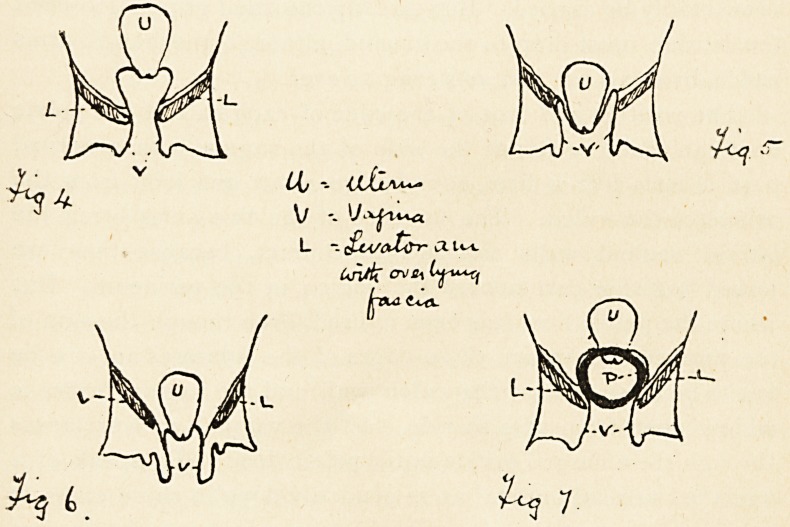


**Fig 8. f5:**
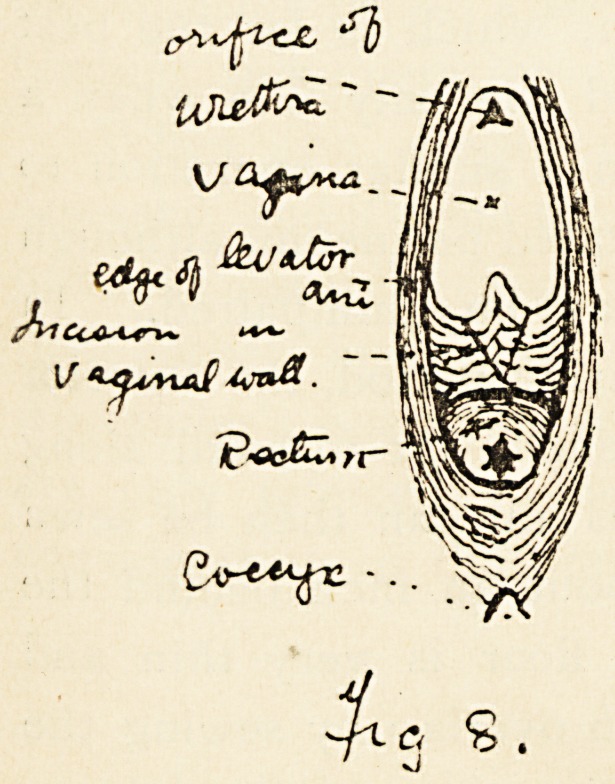


**Fig 9. f6:**